# Metamorphic Buffer Layer Platform for 1550 nm Single-Photon Sources Grown by MBE on (100) GaAs Substrate

**DOI:** 10.3390/ma14185221

**Published:** 2021-09-10

**Authors:** Piotr Andrzej Wroński, Paweł Wyborski, Anna Musiał, Paweł Podemski, Grzegorz Sęk, Sven Höfling, Fauzia Jabeen

**Affiliations:** 1 Technische Physik, University of Würzburg and Wilhelm-Conrad-Röntgen-Research Center for Complex Material Systems, Am Hubland, D-97074 Würzburg, Germany; sven.hoefling@physik.uni-wuerzburg.de (S.H.); fauzia.jabeen@physik.uni-wuerzburg.de (F.J.); 2 Department of Experimental Physics, Faculty of Fundamental Problems of Technology, Wrocław University of Science and Technology, Wybrzeże Wyspiańskiego 27, 50-370 Wrocław, Poland; anna.musial@pwr.edu.pl (A.M.); pawel.podemski@pwr.edu.pl (P.P.); grzegorz.sek@pwr.edu.pl (G.S.); 3 Quantum Light and Matter Group, Faculty of Engineering and Physical Sciences, University of Southampton, Southampton SO17 1BJ, UK

**Keywords:** single-photon source, quantum dots, telecommunication spectral range, metamorphic buffer layer

## Abstract

We demonstrate single-photon emission with a low probability of multiphoton events of 5% in the C-band of telecommunication spectral range of standard silica fibers from molecular beam epitaxy grown (100)-GaAs-based structure with InAs quantum dots (QDs) on a metamorphic buffer layer. For this purpose, we propose and implement graded In content digitally alloyed InGaAs metamorphic buffer layer with maximal In content of 42% and GaAs/AlAs distributed Bragg reflector underneath to enhance the extraction efficiency of QD emission. The fundamental limit of the emission rate for the investigated structures is 0.5 GHz based on an emission lifetime of 1.95 ns determined from time-resolved photoluminescence. We prove the relevance of a proposed technology platform for the realization of non-classical light sources in the context of fiber-based quantum communication applications.

## 1. Introduction

Quantum-dot-based devices required for the implementation of secure communication schemes include, among others, single-photon sources (SPSs), single-photon detectors (SPDs), and quantum repeaters [1,2]. In the approaching age of quantum optoelectronics, it is essential to provide reliable hardware for quantum computing and quantum communication [3], providing a secure information transfer between quantum nodes. The most fundamental component of quantum networks is a source of single photons. It should be characterized by high operation rates and high single-photon purity, but it must also be implemented in the already existing telecommunication network for widespread applications [4]. Therefore, it is desirable for new devices to emit at 1.55 μm, which fits the lowest attenuation window of the optical fibers, offering the longest transmission distance (the telecom C-band).

Several approaches to the growth of InAs quantum dots (QDs) aimed at telecommunication wavelengths have been already reported. The emission in the telecom C-band has been first successfully achieved using InP substrates, owing to larger QDs’ sizes obtained due to lower strain following smaller lattice mismatch in comparison to GaAs-based system [5]. Since then, this approach has been further improved, leading to the observation of single-photon emission [6,7,8,9,10,11,12], (with the record in this spectral range single-photon purity on the level of 4.4 × 10^−4^, obtained under quasi-resonant excitation [13]), also in the electrical excitation scheme [14] and at elevated (liquid nitrogen) temperatures [15,16], with the highest extraction efficiency on the level of 10.9–13% [6,17]. There have also been demonstrated polarization-entangled photons from a light-emitting diode [15]. The InAs/InP system has also allowed for a realization of a quantum key distribution over 120 km [18] and quantum teleportation [19]. Overall, the InP-based structures have been proven as attractive candidates for non-classical light sources, but they still suffer from some intrinsic limitations. For instance, low refractive index contrast of materials lattice-matched to InP complicates the creation of efficient photonic outcoupling, as the design and fabrication of the distributed Bragg reflectors (DBRs) is not straightforward and requires using multinary compounds and a larger number of DBR mirror pairs [20], in contrast to the GaAs-based structures, in which good-quality and high-efficiency Al(Ga)As/GaAs DBRs are well established. In addition, GaAs-based structures benefit from already-developed and fully optimized deterministic nanofabrication and structurization techniques for GaAs-based photonic devices at near infrared [2,3,21,22,23,24] and typically offer a lower spatial density of the optically active QDs, which is crucial for nanophotonic applications. However, reaching the emission in the C-band range with QD structures grown by the molecular beam epitaxy (MBE) on GaAs substrates and realization of corresponding high-efficiency photonic structures remain elusive. Thus far, single-photon emission has been demonstrated only for graded metamorphic buffer layer (MBL)-based QD structures grown on a GaAs substrate by metal–organic vapor phase epitaxy (MOVPE) [24,25]. This approach has been further used to show the emission of entangled [26] or indistinguishable photons [27,28], as well as the possibility of piezo-tuning of the emission energy [29], and the generation of single-photons on demand [30]. However, high-quality photonic cavities containing MBL-based QDs have not been demonstrated yet, which is probably related to the technologically complicated growth that degrades the optical and structural quality of the resulting structure. Originally, MBLs grown by MBE on GaAs substrates were used for laser structures operating in the long-wavelength range (1300–1550 nm) [31]. The structure proposed in [31] is based on an InGaAs MBL, with a fixed In composition, and features threading dislocations arising from the plastic relaxation of the grown material, which strongly degrades optical properties of the QD. High optical quality QDs were only obtained by growing thick enough MBL with constant 22% In content, which resulted in dislocations’ density of 2 × 10^8^ cm^−2^ but limited the emission wavelength to a maximum of 1200 nm. This was later overcome by introducing a linear increase in the In content in the growth direction, resulting in lower strain in the MBL-based structure and hence improving its structural quality by decreasing the number of defects. Furthermore, QDs were capped with an InGaAs layer, which enabled achieving lasing at the third telecom window [31]. However, for these long-wavelength QD emitters, higher dislocations’ density resulted in the decrease of the photoluminescence (PL) intensity by two orders of magnitude. In the case of the laser structure, it was compensated by using multiple QD layers. However, such an approach is inadequate for single QD studies. To the best of the authors’ knowledge, the only attempt to study single QDs in MBE-grown MBL structures uses a complex sample design, with an InGaAs graded buffer and a linear increase in In content, followed by a homogeneous 42% In content MBL, with additional superlattices and InGaAs layers surrounding the QDs [32]. In that study, photoluminescence from single QDs was observed but without any indications of single-photon emission.

In this work, single-photon emission from MBE-grown GaAs-based QDs on an MBL is demonstrated for the first time by comprehensive spectroscopic studies, providing an alternative to the state-of-the-art MOVPE-grown structures [24,25] regarding GaAs-based non-classical light sources operating in the telecom spectral range. We present a modified approach to the MBE-grown MBL by combining the linear grading of the In composition with digital alloying known from laser structures, developed originally for emission around 1 µm [33]. This is achieved by a sub-monolayer deposition of InAs insertions into the InGaAs layer, taking advantage of the sub-monolayer control of material deposition characteristic for MBE growth. It provides the composition gradient and ensures smoother interfaces, without layer thickness fluctuations, needed for high-quality quantum light sources. In comparison to MOVPE, MBE provides better reproducibility and allows a controlled process with sub-monolayer precision of the MBL growth for In contents in a broad range from 6% to 42%, which is essential for implementing such a QD structure design. This is achieved due to a lower number of degrees of freedom in the growth procedure (e.g., in the MOVPE technique, the growth mode depends on the transport of reactants, which are supplied to the sample surface by carrier gases) and more homogeneous growth of the epitaxial material without fluctuations of the layers’ thicknesses.

## 2. Materials and Methods

**Figure 1:**
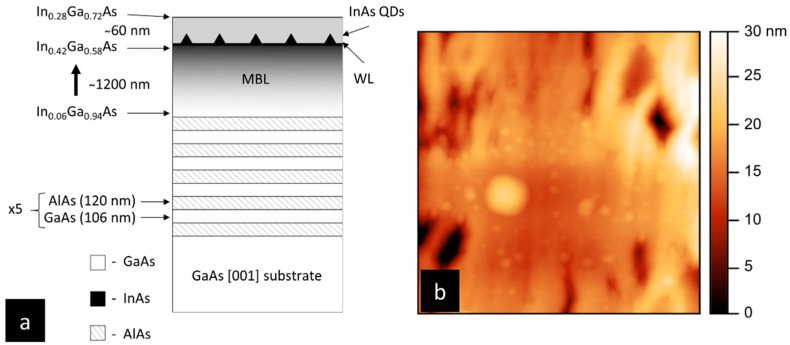
Schematic of the investigated structure and AFM micrograph from a sample grown for morphological analysis: (**a**) from the bottom: GaAs substrate with five DBR pairs, 1200 nm MBL layer where In content increases from 6% to 42%, followed by InAs QDs, capped with 60 nm InGaAs layer containing 28% In; (**b**) a 1 × 1 µm^2^ AFM micrograph with color scale of uncapped structure exhibiting the presence of large 3D island and QDs.

The sample was grown in solid-source MBE on an undoped (100) GaAs substrate. The growth started with deoxidation of the substrate at 630 °C. The substrate temperature was decreased to 580 °C, and a 150 nm thick GaAs buffer was deposited. Five DBR pairs consisting of GaAs/AlAs layers with 106 nm and 120 nm thickness were grown. The substrate temperature was decreased to 460 °C, and a thin GaAs layer was grown. For the growth of the MBL, we combined linear grading of the In composition with sub-monolayer deposition of InAs insertions into the InGaAs layer (digital alloying) [33]. Growth rates of 40 nm/h and 560 nm/h were used for InAs and GaAs, respectively. InGaAs ternary material with 6% In and 30 nm thickness was grown as a seeding layer. In the next step, 0.05 Å InAs was inserted in In_0.06_Ga_0.94_As ternary to obtain 7% In content. This formed a 0.4 nm period, which was repeated till a stack with a total thickness of 30 nm was formed. For subsequent stacks, the width of InAs insertion was increased, while stack width per indium step was kept constant. After the growth of 21% In containing stack, the substrate temperature was decreased to 450 °C. In content was increased up to 42% following the abovementioned sub-monolayer InAs insertions in formerly obtained InGaAs ternary compound. This resulted in a total thickness of the MBL layer of 1200 nm. QDs were grown in the Stranski–Krastanow growth mode. For QDs, an InAs layer with a nominal thickness of 1.5 ML was deposited and followed by a 60 nm thick In_0.28_Ga_0.72_As layer to cap the QDs (Figure 1a). An analysis of atomic force microscopy (AFM) (Anfatec Instruments AG, Oelsnitz, Germany) images of the uncapped QDs shows two distinct QD families: large islands with 200 nm diameter (most probably not optically active or, even if so, with the expected emission out of the spectral range of interest) and QDs with diameters in the range of 20–50 nm and a size distribution typical for the self-assembled growth. A 1 × 1 µm^2^ AFM image of an exemplary spot on the sample surface is shown in Figure 1b. The density of all nano-objects on the sample surface is estimated to be in the range of 10^10^ cm^−2^, with significant QDs’ density non-uniformity present across the wafer area, with some areas featuring much lower spatial density, actually preferred for the single-dot study. The number of emission lines originating from distinct QDs observed in the photoluminescence experiment in the target spectral range of the 3rd telecom window corresponds to optically active QDs’ density of about 10^9^ cm^−2^. In addition to QDs, one can observe parallel longitudinal pits on the sample surface. These are the signature of threading dislocations propagating within the MBL toward the surface of the sample. Although Indium content change is modulated, such threading dislocations can emerge from lattice change during In content increase due to relaxation from build-up strain. Improvement of the MBL growth parameters is needed to further suppress them.

For all the optical measurements the sample was kept in a liquid-helium continuous-flow cryostat at the temperature of about 5 K. The optical characterization of QDs’ emission was performed using a microphotoluminescence (µPL) setup based on a 1 m focal-length spectrometer (HORIBA, Kyoto, Japan, FHR 1000), coupled with a liquid-nitrogen-cooled InGaAs linear array detector (HORIBA, Kyoto, Japan, Symphony II) offering effective spectral resolution better than 25 µeV. The sample was excited using a continuous-wave (cw) 640 nm laser line focused on the sample surface by a long working distance objective with NA = 0.4 to a beam diameter of about 1–2 µm. In order to determine the polarization properties of emission, a rotating half-wave plate and a fixed linear polarizer were placed in front of the monochromator entrance slit. Time-resolved photoluminescence (TRPL), by means of time-correlated single-photon counting, and photon statistics using Hanbury Brown and Twiss configuration were measured using 0.32 m focal-length monochromator as a spectral filter (0.43 nm bandwidth) (HORIBA, Kyoto, Japan, IHR320) for selection of a single QDs’ emission lines. For TRPL, we employed an 805 nm semiconductor diode laser with an 80 MHz pulse train and approximately 50 ps long pulses, whereas for autocorrelation measurements, cw excitation with a 787 nm laser line was used. These measurements were performed with fiber-coupled NbN superconducting nanowire single-photon detectors (SCONTEL, Moscow, Russia) with approximately 80% quantum efficiency and dark counts rate of 100 cps at 1.55 µm, connected with a multichannel picosecond event timer. The overall temporal resolution of the experimental setup was 80 ps.

## 3. Results and Discussion

**Figure 2:**
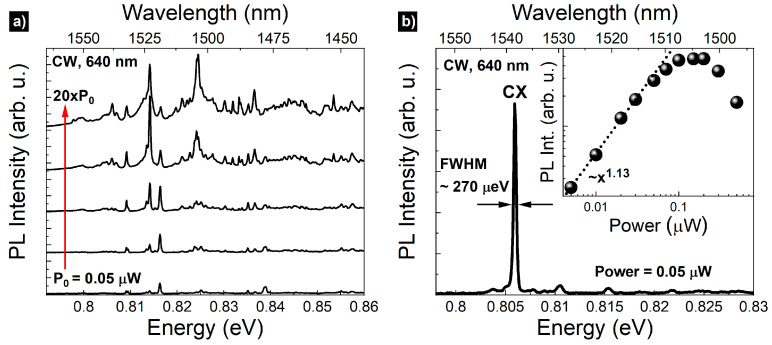
(**a**) Broad range µPL spectrum from planar structure as a function of excitation power from 0.05 µW to 1 µW (red arrow); (**b**) µPL spectrum from a single QD under cw excitation (640 nm). Inset: intensity of the charged exciton (CX) as a function of the excitation power with the power function fit (dotted line).

**Figure 3:**
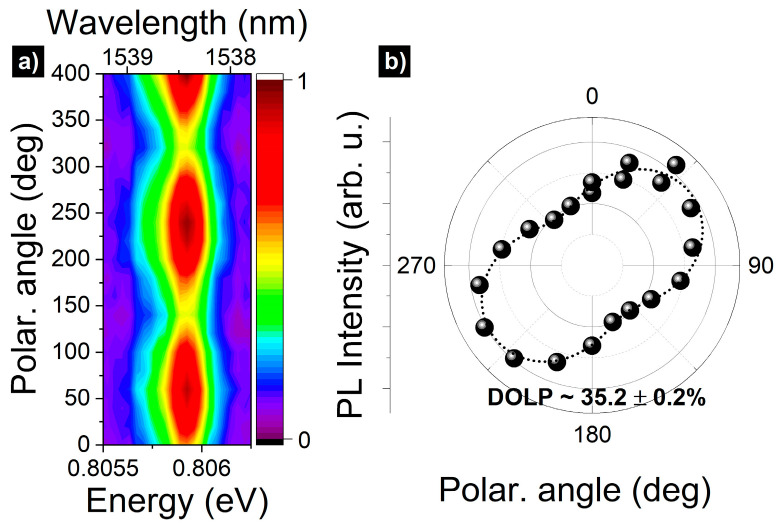
(**a**) Polarization-resolved µPL spectral map for CX emission; (**b**) the intensity of µPL polarization for CX line. The dashed line is a fit (Equation (1)) to the experimental data, indicating approximately 35% of the DOLP value.

Excitation power-dependent broad range µPL spectrum from a planar (unpatterned) structure measured under 640 nm cw non-resonant excitation at low temperature (T~5 K) is presented in Figure 2a. The spectrum shows sharp emission lines from single QDs in the spectral range of the third telecom window. The spatial density of the QDs is low enough to resolve single emission lines. In order to have the possibility of systematic and repeatable single QD studies, we fabricated circular apertures in a thin silver layer additionally deposited on the surface of the structure. Figure 2b shows a spectrum in the C-band range through an 1800 nm diameter aperture (laser excitation power of 0.05 µW, measured outside of the cryostat). The strongest, well-isolated single line, centered at 0.8059 eV (1538.42 nm), is selected for further single-photon emission study. Excitation power dependence of its PL intensity (inset in Figure 2b) exhibits a close-to-linear dependence, with a saturation of emission intensity starting at approximately 0.1 µW. Additionally, polarization independence of the emission energy, shown in Figure 3a, shows a lack of a fine structure splitting (FSS), indicating a probable origin of the line, i.e., radiative recombination of a charged exciton (CX). The linewidth of this CX line is about 270 µeV and stems mostly from the spectral diffusion processes predominant for a non-resonant excitation scheme [34].

Figure 3b shows a dependence of the µPL intensity on the linear polarization angle. Based on that, we can determine the degree of linear polarization: $\mathrm{DOLP}=\left(I_{max}-I_{min}\right)/\left(I_{max}+I_{min}\right)$. These data were fitted to estimate DOLP value by using the following function [35]:

(1) I(θ)=A(1+DOLP⋅cos(2(θ−ϕ)))

where I(θ) is the intensity of µPL as a function of the polarization angle θ, A is the scaling factor, and ϕ is an offset from the 0° angle. Here, the DOLP value is about 35%, which is unusually large for GaAs-based QDs grown on (100) surface [36]. It can be associated with the significant mixing of light-hole (LH) and heavy-hole (HH) states, and it is a fingerprint of anisotropy in the system. It is difficult to resolve which factor has the dominant contribution to the asymmetry of the confinement potential of QDs, i.e., the dots shape asymmetry or residual strain anisotropy originating from the metamorphic buffer layer [36,37,38]. It cannot be judged unequivocally based on the dots geometry seen in the AFM images (where the dots seem rather symmetric), because the buried dots, which are optically active, can differ in morphology from the surface ones [39]. Additionally, significant dot-to-dot shape and strain variations are expected in such an inhomogeneous system [37]. The exact origin of the high DOLP value (related to details of both, morphology and strain) is beyond the scope of this work and will be a subject of an independent report. Typically, the large polarization anisotropy should also translate into a significant value of the fine structure splitting (FSS) for neutral exciton states. However, the investigated QD emission does not show any FSS within the resolution of our optical setup (25 µeV), which is an additional argument for the charged exciton origin of this particular line.

**Figure 4:**
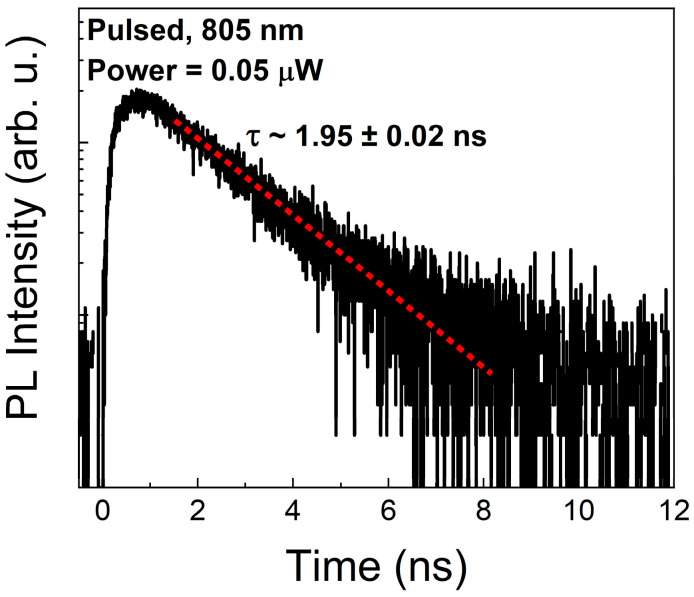
The time-resolved µPL trace for the CX line at low excitation power of 0.05 µW. The red dotted line indicates a mono-exponential fit to the experimental data.

TRPL measurements were carried out to characterize the emission kinetics, in particular, the µPL lifetime limiting the emission rate of the final SPS. The TRPL trace obtained for the selected CX line is presented in Figure 4. The photoluminescence lifetime determined from a fit with a mono-exponential decay (red dotted line in Figure 4) is (1.95 ± 0.02) ns, which is close to the values reported for other InAs QD-like structures on GaAs substrate [27]. The measurement was performed at low excitation power (0.05 µW) in order to minimize the state-filling effects affecting carrier dynamics at higher pumping and to determine the characteristic time constant being close to the radiative lifetime of the charged exciton state. The obtained value can be directly related to the fundamental upper limit for the single-photon emission rate, which is approx. 0.5 GHz in our case, i.e., when assuming the maximum extraction efficiency of emission, 100% internal quantum efficiency, and no additional losses of carriers inside the structure.

**Figure 5:**
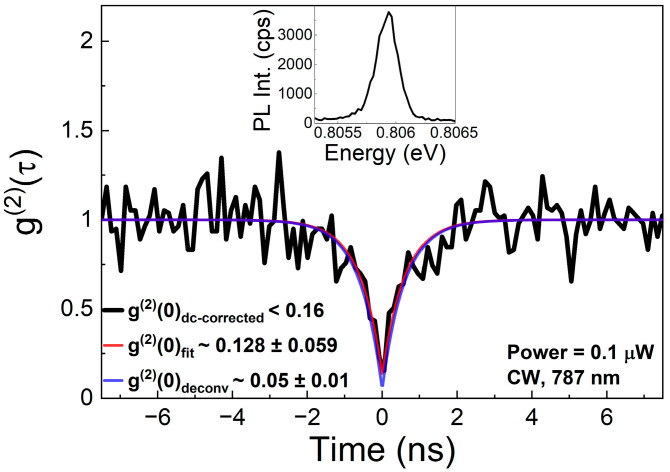
Second-order correlation function g^(2)^(τ) for CX emission under continuous-wave excitation after correction based on dark counts on detectors (black line), direct fit with the Equation (2) (red line), and after the histogram deconvolution with the setup response function (blue line). Inset: corresponding µPL spectrum from the correlation setup for the excitation power of 0.1 µW.

Single-photon emission properties of the investigated GaAs-based QDs were determined by measuring the second-order correlation function g^(2)^(τ) under non-resonant cw excitation (787 nm)—see Figure 5. The inset shows the corresponding µPL spectrum. The obtained antibunching behavior with the as-measured g^(2)^(0) value below 0.21 proves the single-photon character of emission. The experimental g^(2)^(τ) function has been further corrected for the detector dark counts (g^(2)^(0) value below 0.16) and fitted by the following function:

(2) $$g^{\left(2\right)}\left(\tau \right)=1-\left(1-g^{\left(2\right)}\left(0\right)\right)e^{\frac{\left|\tau \right|}{t_{\mathrm{rise}}}}$$

resulting in the value of g^(2)^(0) approx. 0.128 ± 0.059. Moreover, after taking into account the finite temporal resolution of the experimental setup (histogram deconvolution with the setup response function), the final g^(2)^(0) value is 0.05 ± 0.01.

This value of g^(2)^(0), in spite of being measured for still an unoptimized structure design and growth conditions, is comparable with the single-photon purity obtained for InAs/InGaAlAs/InP quantum dashes emitting at 1.55 μm [9]. For variety of InAs/InP or InAs/InGaAslAs/InP nanostructures (both symmetric and asymmetric) values spanning from 0.2 to close to zero were obtained under non-resonant excitation [6,10,12,17,40,41]. The lowest probability of multiphoton events reported for the GaAs-based MOVPE-grown MBL approach yielded g^(2)^(0) = 0.003 ± 0.137 [24] (non-resonant cw excitation), showing that at least an order of magnitude improvement should be reachable after the structure optimization. The record value for the 1.55 μm spectral range among all the single-photon emitters (not only QD-based)—(4.4 ± 0.2) × 10^−4^—achieved for InAs/InP QDs under quasi-resonant pumping [13], points at the importance of optimizing the excitation scheme as well, to achieve the best single-photon source performance.

## 4. Conclusions

In this work, we presented the optical properties of InAs QDs grown by MBE on a GaAs substrate, with the use of a digitally alloyed MBL with graded In content to achieve a good crystalline quality of QDs emitting in the application-relevant telecommunication C-band. Optical characterization showed sharp emission lines originating from single QDs in the desired spectral range. The excitation-power-dependent and the linear-polarization-resolved PL spectra allowed for the identification of a selected bright line as originating from the radiative recombination of a charged exciton. The measured second-order correlation function showed a clear antibunching dip, with the as-measured value at the zero time delay below 0.21 and a value of 0.05 after the dark count correction and deconvolution, confirming a single-photon character of emission from such dots. This demonstrates the potential of the MBE-grown, GaAs-based MBL platform for QD-based single-photon sources in the telecom spectral range, as an alternative to MOVPE-grown structures [24].

## Data Availability

The data that support the findings of this research are available from the corresponding author upon reasonable request.
